# Supplementary material for: The Differential Effect of Senolytics on SASP Cytokine Secretion and Regulation of EMT by CAFs
Source: Int J Mol Sci. 2024 Apr 4;25(7):4031. doi: 10.3390/ijms25074031 (PMC11012227; doi:10.3390/ijms25074031)
Supplement: Supplementary file 1 [file ijms-25-04031-s001.zip › ijms-2939084-supplementary.pdf]

Supplementary Table S1. Primer sequences on mouse genes.

Name	Sequence
m-p16-F	CGTGAACATGTTGTTGAGGC
m-p16-R	GCAGAAGAGCTGCTACGTGA
m-p21-F	CAGATCCACAGCGATATCCA
m-p21-R	ACGGGACCGAAGAGACAAC
mFap-F	CCGCGTAACACAGGATTCACTG
mFap-R	CACACTTCTTGCTCGGAGGAGA
m-α-Sma-F	CCCCTGAAGAGCATCGGACA
m-α-Sma-R	TGGCGGGGACATTGAAGGT
mZeb1-F	ATTCAGCTACTGTGAGCCCTGC
mZeb1-R	CATTCTGGTCCTCCACAGTGGA
mSnail-F	TGTCTGCACGACCTGTGGAAAG
mSnail-R	CTTCACATCCGAGTGGGTTTGG
mIl6 F	TACCACTTCACAAGTCGGAGGC
mIl6 R	CTGCAAGTGCATCATCGTTGTTC
mGapdh F	TGGTGAAGGTCGGTGTGAAC
mGapdh R	CCGTTGAATTTGCCGTGAGT
mIl10-F	TACAGCCGGGAAGACAATAA
mIl10-R	AGCAGTCGGTTAGCAGTATG
mIl-1A-F	AAGTCTCCAGGGCAGAGAGG
mIl-1A-R	CTGATTCAGAGAGAGATGGTCAA
mIl-1B-F	AAAAGCCTCGTGCTGTCG
mIl-1B-R	AGGCCACAGGTATTTTGTCG
